# Doxorubicin-loaded gold nanoparticles for enhanced anticancer efficacy: in vitro, in vivo, and FTIR-based tissue analysis

**DOI:** 10.1038/s41598-026-61018-3

**Published:** 2026-07-25

**Authors:** Amna H. Faid, Ali Abdelaziem, Nehal Ali, Fatma El Zahraa Hussein, Sara Gad, Marwa Sharaky, Heba N. Deif

**Affiliations:** 1 Department of Laser Sciences and Interactions, National Institute of Laser Enhanced Science (NILES), Cairo University, Giza, 12613 Egypt; 2 Department of Engineering Physics and Mathematics, Faculty of Engineering, Tanta University, Tanta, Egypt; 3 Faculty of Engineering, Helwan University, Cairo, 11795 Egypt; 4 Electronic Materials Research Department, Advanced Technology and New Materials Research Institute, City of Scientific Research and Technological Applications (SRTA-City), Alexandria, Egypt; 5 National Cancer Institute (NCI), Cairo University, Cancer biology, Giza, 11796 Egypt; 6 Microbiology Department, Faculty of Veterinary Medicine, Cairo University, Giza, Egypt

**Keywords:** Cancer nanomedicine, Doxorubicin delivery, Ehrlich carcinoma, FTIR spectroscopy, Gold nanoparticles, In vitro and in vivo evaluation, Nanobiotechnology, Tumor tissue fingerprinting, Biotechnology, Cancer, Chemistry, Drug discovery, Materials science, Nanoscience and technology

## Abstract

The most challenging aspects of cancer treatment are multi-drug resistance (MDR) and damage to normal, non-malignant cells. Doxorubicin (Dox)-loaded gold nanoparticles (Dox@AuNPs) were developed and evaluated for enhanced anticancer activity. The nanocomposites exhibited spherical morphology (13 ± 3 nm) with a loading efficiency of 52%. In vitro, Dox@AuNPs reduced the IC₅₀ by approximately 50% compared to free Dox in MCF-7 cells. In vivo, treatment significantly suppressed tumor growth and increased median survival (62 days) relative to free Dox (52 days). In addition to conventional efficacy evaluation, Fourier transform infrared (FTIR) spectroscopy was applied to analyze biochemical changes in tumor tissues. Distinct alterations in protein, lipid, and nucleic acid-associated bands were observed, with treated tissues showing partial spectral shifts toward normal profiles. These findings suggest that FTIR may provide additional insight into tissue-level biochemical responses following treatment. While further validation and toxicity assessment are required, the results demonstrate that Dox@AuNPs enhance anticancer efficacy and highlight the potential utility of FTIR as a complementary tool for evaluating therapeutic response.

## Introduction

In 2024, approximately 2,001,140 new cancer cases and 611,720 cancer deaths are projected in the United States^1^, with breast cancer accounting for 10% of newly diagnosed cases and representing the second leading cause of female cancer mortality^2,3^. Conventional chemotherapeutic agents, such as doxorubicin (Dox), are limited by poor solubility, short circulation half-life, non-specific targeting, and dose-dependent cardiotoxicity^4–8^. Nanomedicine provides promising strategies to overcome these challenges by enabling targeted drug delivery, reducing systemic toxicity, and enhancing therapeutic efficacy^9–12^. Among various nanocarriers, gold nanoparticles (AuNPs) are attractive due to their high biocompatibility, controlled size, facile surface functionalization, and stability, making them ideal candidates for Dox delivery^13–15^ when compared to alternative nanostructures such as silver or carbon-based materials. In contrast to organic liposomes, which may experience early cargo leakage, or magnetic nanoparticles that can pose toxicity risks at elevated concentrations, gold is chemically stable and exhibits low immunogenicity in both murine and human models. A significant technical advantage lies in the simplicity of modifying their surface; through gold-thiol chemistry, researchers can attach a high density of therapeutic agents (such as doxorubicin), targeting ligands (including folic acid or antibodies), and PEGylation layers to prolong systemic circulation time and leverage the Enhanced Permeability and Retention (EPR) effect^16^.

Several studies have explored Dox-loaded nanoparticles for cancer therapy, showing improved cytotoxicity and tumor inhibition. However, most investigations focus on in vitro cytotoxicity or in vivo tumor volume reduction, with limited evaluation of tissue-level biochemical responses^17,18^.

Fourier transform infrared (FTIR) spectroscopy has been widely used to characterize chemical interactions in nanocomposites but remains underutilized for assessing therapeutic effects directly in tumor tissues. The method of FTIR spectroscopy has been utilized in optical tissue diagnostics to analyze and characterize the structure of numerous substances, including polypeptides and proteins^19^. Fourier-Transform Infrared (FTIR) spectroscopy has emerged as a high-precision diagnostic modality in oncological research, capable of distinguishing between normal, malignant, and post-treated tissues by isolating specific biochemical “fingerprints”^20^. In differentiating normal from malignant tissue, recent studies have utilized FTIR to detect intrinsic biomarkers of carcinogenesis, such as significant shifts in protein secondary structures (amide I and III regions) and alterations in the cytoplasm-to-nucleus ratio^21^. Beyond initial diagnosis, FTIR is increasingly critical for assessing treatment efficacy. Post-treated tissues exhibit unique spectral signatures associated with cellular recovery or therapy-induced stress; for instance, identifying the “spectral distance” between treated tumors and healthy control tissue allows researchers to quantify the extent of molecular normalization^22^.

In this work, the preparation, characterization of Dox@AuNPs nanocomposites, and their anticancer efficacy in MCF-7 breast cancer cells and Ehrlich carcinoma-bearing mice were reported and evaluate. Importantly, FTIR spectroscopy to tumor tissues post-treatment was applied to assess biochemical changes, providing a mechanistic insight into therapeutic response and tissue normalization. This integrative approach highlights a novel strategy to evaluate nanoparticle-based chemotherapy at both cellular and tissue levels.

## Materials and methods

### Preparation of AuNPs

AuNPs were synthesized by the citrate reduction of HAuCl₄ as previously described^23^. Briefly, 50 mL of 1 mM HAuCl₄ was heated to boiling, then 5 mL of 38.8 mM sodium citrate was rapidly added under vigorous stirring. The solution color changed from bright yellow to red, indicating formation of AuNPs^23^.

### Preparation of Dox@AuNPs Nanocomposites (NCs)

Dox@AuNPs NCs were prepared by incubating 1 mL of AuNPs with 1 mL of varying Dox concentrations (0.001-1 mM) under continuous stirring and sonication for 15 min, followed by incubation at room temperature for 24 h. Unbound Dox was removed by centrifugation at 12,000 rpm for 30 min^24,25^.

### Characterization of AuNPs and Dox@AuNPs NC

UV-Vis spectroscopy (200–800 nm), TEM, dynamic light scattering (Zetasizer), fluorescence spectroscopy, and FTIR (Shimadzu 8400, 500–4500 cm⁻¹) were used to characterize morphology, size, surface charge, optical properties, and drug-nanoparticle interactions. Lyophilized samples were prepared for FTIR using KBr pellets at 1:100 sample-to-KBr ratio.

### Dox loading efficiency

Loading efficiency (%) was calculated indirectly by measuring free Dox in the supernatant using UV-Vis spectroscopy at 420 nm^8^ as follows:

1 $$\:\mathbf{D}\mathbf{O}\mathbf{X}\:\mathbf{l}\mathbf{o}\mathbf{a}\mathbf{d}\mathbf{i}\mathbf{n}\mathbf{g}\:\mathbf{e}\mathbf{f}\mathbf{f}\mathbf{i}\mathbf{c}\mathbf{i}\mathbf{e}\mathbf{n}\mathbf{c}\mathbf{y}\:\left(\mathbf{\%}\right)=\left(\frac{\mathrm{T}\mathrm{o}\mathrm{t}\mathrm{a}\mathrm{l}\:\mathrm{D}\mathrm{O}\mathrm{X}-\mathrm{F}\mathrm{r}\mathrm{e}\mathrm{e}\:\mathrm{D}\mathrm{O}\mathrm{X}}{\mathrm{T}\mathrm{o}\mathrm{t}\mathrm{a}\mathrm{l}\:\mathrm{D}\mathrm{O}\mathrm{X}}\times100\right)\:$$

### In vitro cytotoxicity (SRB assay)

MCF-7 cells (ATCC) were cultured in RPMI-1640 with 10% FBS and 2% penicillin/streptomycin at 37 °C, 5% CO₂. Cells were seeded at 5 × 10³ cells/well in 96-well plates, allowed to adhere for 24 h, and treated with Dox or Dox@AuNPs NCs (0.1–80 µM) for 24–72 h. Cell viability was assessed using SRB assay as previously described^26^ IC₅₀ values were calculated from triplicate experiments.

2 $$\:\mathbf{S}\mathbf{u}\mathbf{r}\mathbf{v}\mathbf{i}\mathbf{v}\mathbf{a}\mathbf{l}\:\mathbf{f}\mathbf{r}\mathbf{a}\mathbf{c}\mathbf{t}\mathbf{i}\mathbf{o}\mathbf{n}=\frac{(\mathrm{O}.\mathrm{D}.\:(\mathrm{t}\mathrm{r}\mathrm{e}\mathrm{a}\mathrm{t}\mathrm{e}\mathrm{d}\:\mathrm{c}\mathrm{e}\mathrm{l}\mathrm{l}\mathrm{s})}{(\mathrm{O}.\mathrm{D}.\:(\mathrm{c}\mathrm{o}\mathrm{n}\mathrm{t}\mathrm{r}\mathrm{o}\mathrm{l}\:\mathrm{c}\mathrm{e}\mathrm{l}\mathrm{l}\mathrm{s})}\:$$

### In vivo antitumor efficacy

Ehrlich ascites carcinoma (EAC) cells (2 × 10⁶) were injected subcutaneously into female Swiss albino mice. Mice were randomly divided into four groups (*n* = 10): saline control, AuNPs, free Dox (3.74 mg/kg), and Dox@AuNPs NC (equivalent Dox dose). Tumor volume was measured twice weekly using calipers (V = 0.52 × a² × b, a = width, b = length). Survival and body weight were monitored. Tumor tissues were collected for FTIR analysis post-treatment^27,28^. At the end of the experiment, mice were euthanized by cervical dislocation performed by trained personnel in accordance with institutional animal care and ethical guidelines. No chemical anesthesia or euthanasia agents were administrated.

### FTIR analysis of tumor tissue

Lyophilized tumor tissue was mixed with KBr (1:100), pressed into pellets, and scanned (400–4000 cm⁻¹) using an FTIR spectrometer. Spectral differences between normal, malignant, and treated tissues were analyzed to assess biochemical changes^29,30^.

### Statistical analysis

Data are presented as mean ± SD. Statistical significance was determined using one-way ANOVA with Tukey’s multiple comparison test; *p* ≤ 0.05 was considered significant. Analyses were performed using GraphPad Prism v5.

## Results

### Characterization of AuNPs and Dox@AuNPs NC

**Fig. 1:**
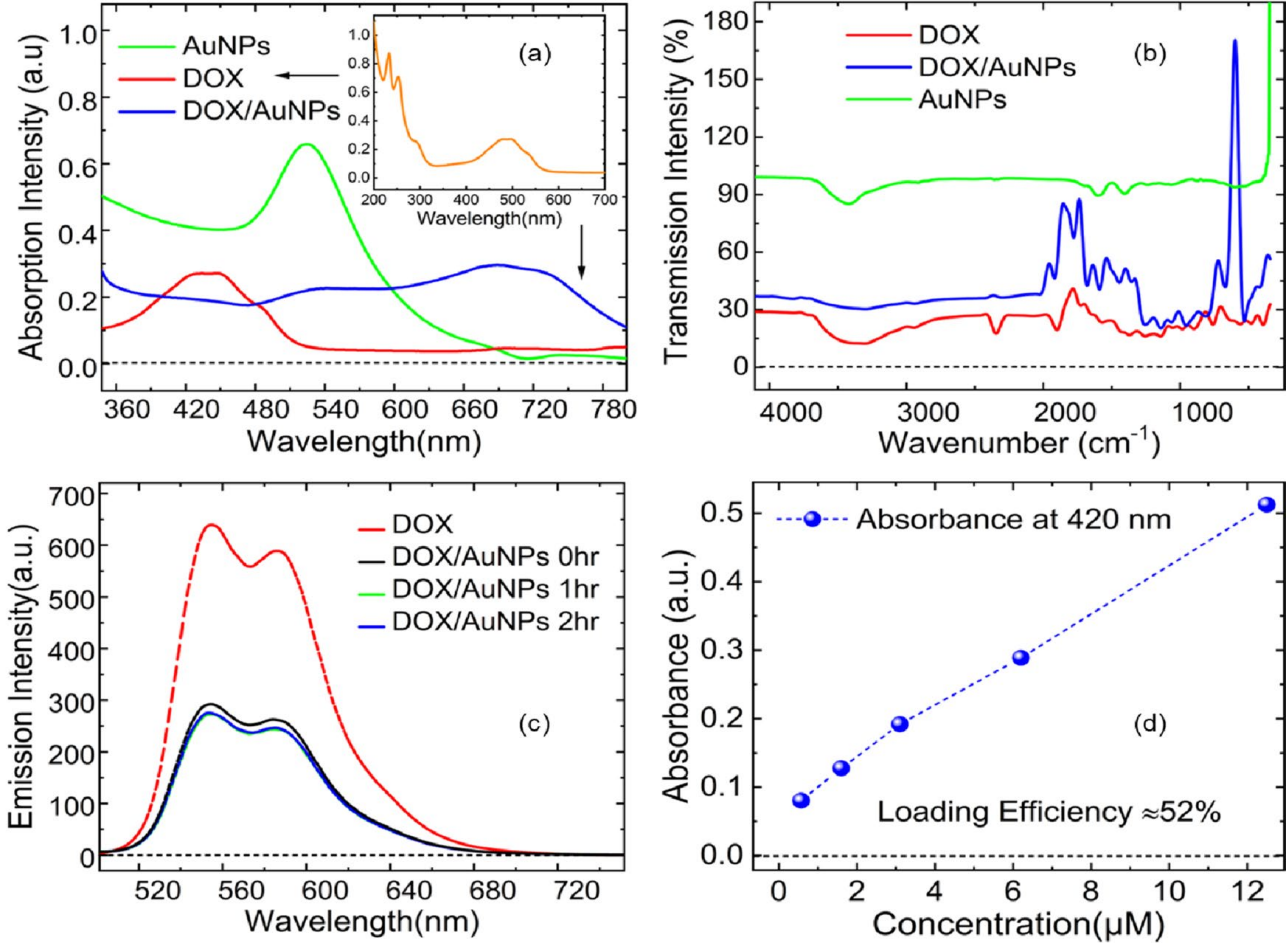
Optical properties of the synthesized samples. (**a**) UV/VIS spectra of AuNPs, Dox, Dox@AuNPs NC; (**b**) FTIR spectra; (**c**) Emission spectra; (**d**) UV calibration curve.

**Fig. 2:**
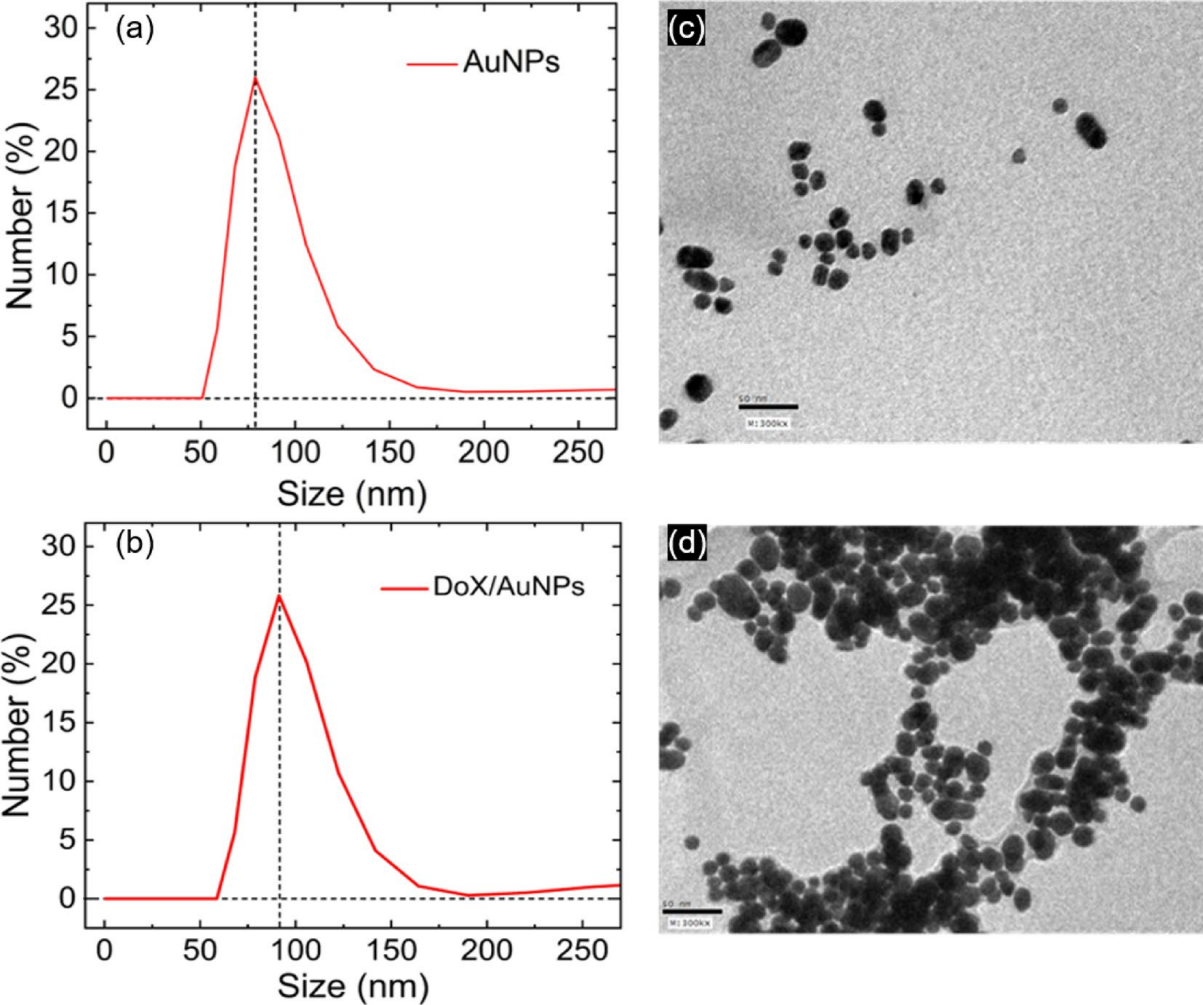
(**a**) Zeta sizer of AuNPs, (**b**) Zeta sizer of Dox@AuNPs NC, (**c**) TEM image of AuNPs, and (**d**) TEM image of Dox@AuNPs NC.

AuNPs displays an absorption peak due to surface plasmon resonance (SPR) at 521 nm. Dox displays a band covering from 400 nm to 500 nm with a peak at about 420 nm as shown in Fig. 1a. This band belongs to anthracyclines. The size of AuNPs and Dox@AuNPs NC was analyzed using Zeta sizer analysis as shown in Fig. 2a; the average size of AuNPs and the DOX-AuNPs nanocomposite was about 70 nm and 95 nm respectively. TEM images of AuNPs showed spherical particles with a size of 12 ± 4 nm as seen in Fig. 2b. TEM images of Dox@AuNPs NC; show that the nanocomposite has a spherical shape with a minor increase in the particle size from 12 ± 4 nm to 14 ± 4 nm as seen in Fig. 2c the size increase corresponds to the replacement of citrate with Dox. Fluorescence investigations are an ideal probe for validating drug binding to AuNPs because Au metal effectively quenches the emission of numerous fluorophores, as previously documented. Figure 1c shows that the spectral profile of Dox@AuNPs NC did not change significantly, and the bands at 540 and 580 nm that are detected for Dox were preserved in the presence of AuNPs. The binding of Dox@AuNPs NC was additionally examined using FTIR studies. The IR spectra of AuNPs, free Dox, and Dox@AuNPs NC are illustrated in Fig. 1b. AuNPs exhibited two peaks at 3450 and 1635 cm^− 1^ and a middle band at 1265 cm^− 1^. The broad peak at 3400 cm^− 1^ associates with O–H stretching vibrations owing to intermolecular hydrogen bonding. While peaks at 1630 and 1265 cm^− 1^ correspond to –C = O stretching and C–O stretching respectively.

The gradual decrease in fluorescence intensity over time suggests progressive interaction and binding of Dox molecules to the Au nanoparticles. This fluorescence quenching may indicate successful drug loading and conjugation onto the nanoparticle surface. The observed fluorescence quenching indicates successful drug loading and conjugation onto the nanoparticles. Monitoring fluorescence over time provides insight into the kinetics of the loading process, demonstrating that Dox-AuNP interaction is time dependent rather than instantaneous. The reduction in fluorescence intensity over time may be attributed to an increasing proportion of Dox molecules becoming associated with the AuNP surface, resulting in decreased fluorescence from free Dox in solution.

### Loading efficiency

The loading efficiency was calculated from the UV spectrum as shown in Fig. 1d. The standard curve of Dox was obtained by plotting concentrations from 0.57 to 12.5 *µM* against their respective absorbance at 420 nm, Upon addition of Dox to AuNPs, the band intensity decreased which may be due to a reduction in Dox concentration which confirmed its loading onto the AuNPs surface. The loading efficiency was found to be 52%.

### In vitro cytotoxicity

**Fig. 3:**
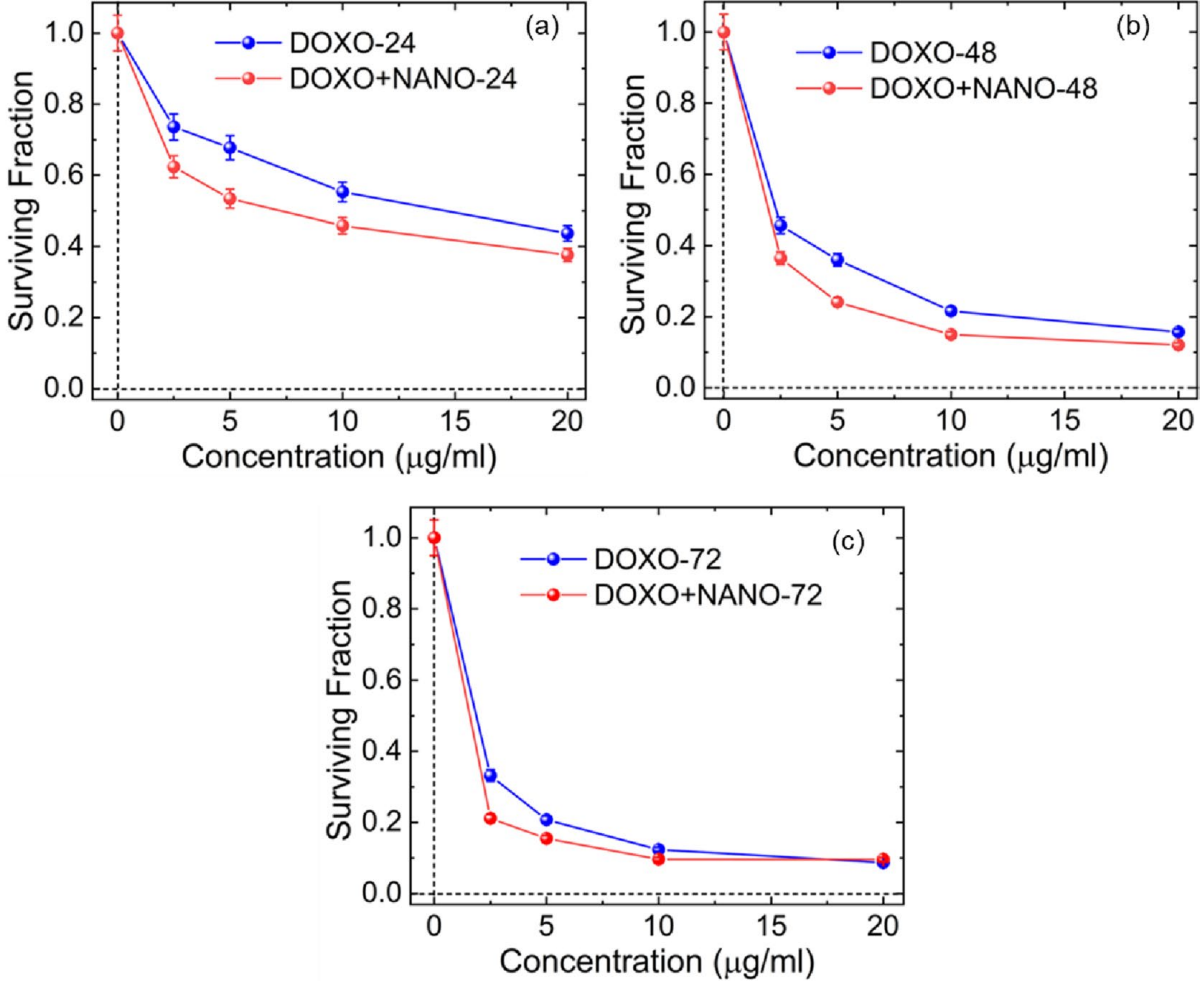
Cytotoxicity of Dox and Dox@AuNPs NC on MCF7 at 24 h., 48 h. and 72 h.

The cytotoxic effect of different concentrations of Dox and Dox@AuNPs NC (5, 0.1, 0.2, 0.4, and 0.8 µM) on the breast cancer cell line (MCF-7) after 24 h, 48 h, and 72 h is shown in (Figure 3a, b, and c), respectively. The results indicate that both concentration- and time-dependent decreases in cell proliferation were observed compared to the control. Dox@AuNPs NC resulted in decreased cell viability, with an IC₅₀ approximately half that of free Dox.

### In vivo antitumor activity and survival

**Fig. 4:**
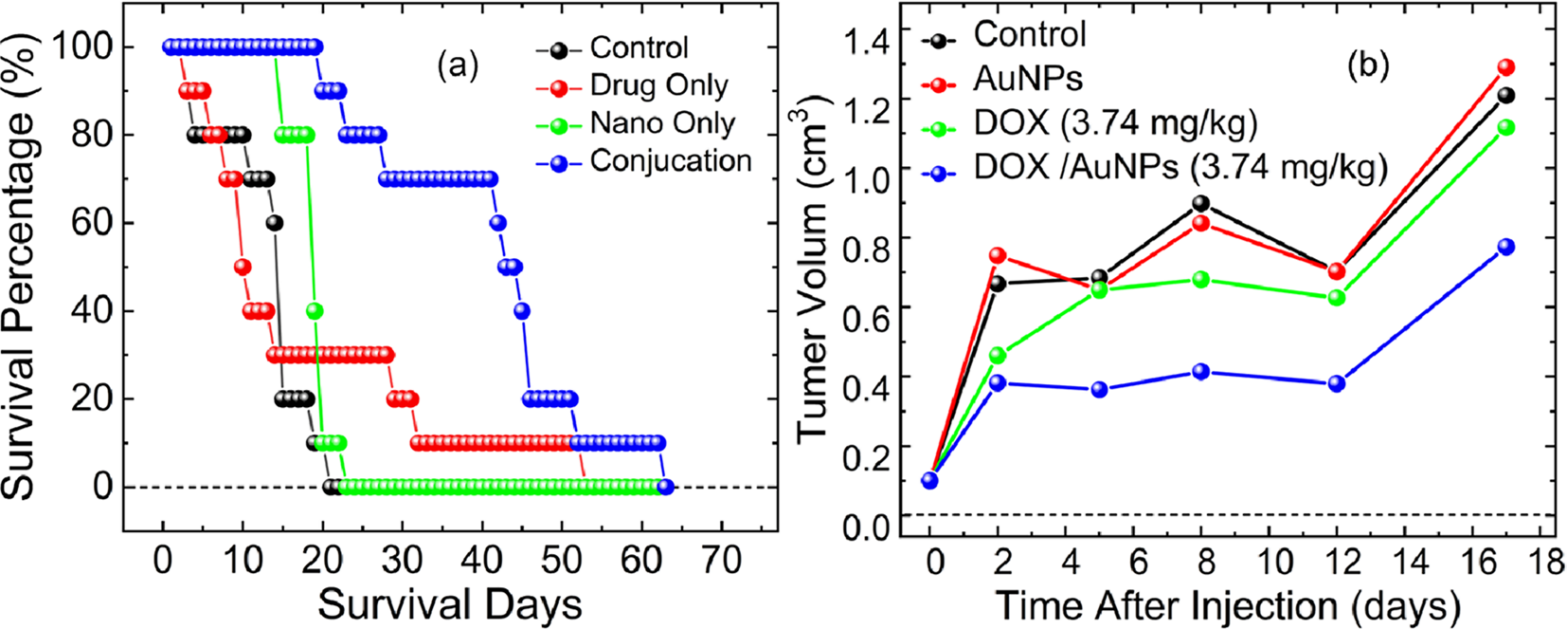
(**a**) Kaplan-Meier survival curves of mice in different treatment groups. (**b**) Tumor volume changes over time following different treatments.

Ehrlich carcinoma is considered highly susceptible to chemotherapy due to its similarity to human tumors. The in vivo efficacy of the nanocomposite was evaluated by monitoring tumor volume and survival after intraperitoneal administration. Dox@AuNPs NC treatment resulted in a statistically significant reduction in tumor growth compared with free Dox and AuNPs alone (*p* ≤ 0.05) (Fig. 4b). As shown in Fig. 4a, median survival was 62 days for the Dox@AuNPs NC group, 52 days for free Dox, 22 days for AuNPs, and 20 days for controls. Overall, Dox@AuNPs NC significantly inhibited tumor growth and improved survival compared with all other groups.

### FTIR analysis of tumor tissue

**Fig. 5:**
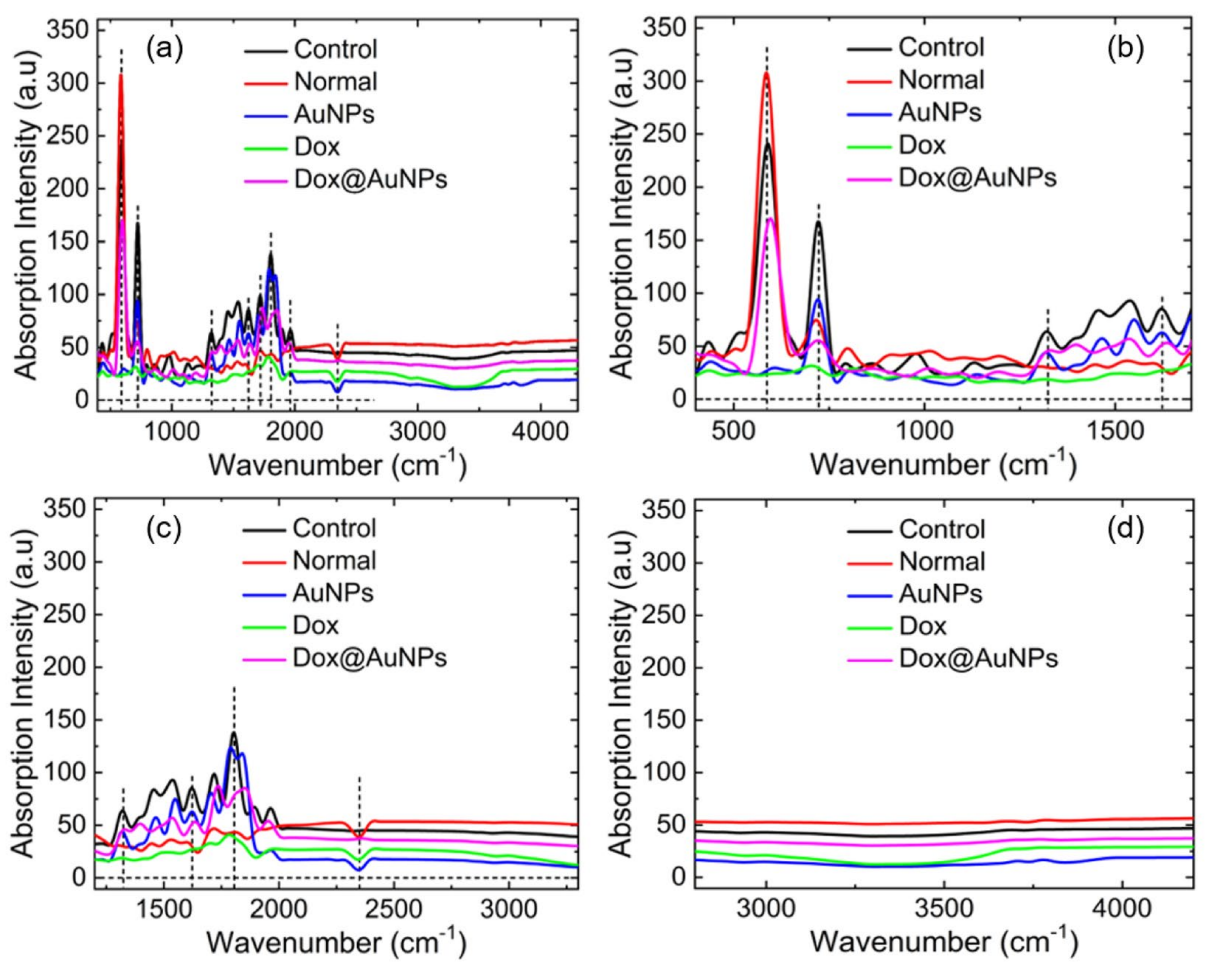
FTIR spectra (**a**, **b**, **c**, and **d**) at different wavenumber ranges (cm⁻¹) for control, normal, AuNPs, malignant, and Dox@AuNPs NC-treated tissues. Data are presented as mean ± SD, and statistical significance was analyzed using ANOVA (*p < 0.05, **p < 0.01).

FTIR spectra revealed distinct differences between normal and malignant tissues. Following Dox@AuNPs NC treatment, spectra shifted toward normal tissue, particularly in nucleic acid (1077 cm⁻¹, 1261 cm⁻¹), protein (amide I, II, III), lipid (CH₂/CH₃, 2900–3100 cm⁻¹), collagen (1034 cm⁻¹), and glycogen (1151 cm⁻¹) bands (Fig. 5). These spectral shifts are consistent with partial restoration of biochemical composition in tumor tissue.

### Statistical analysis

All experiments were performed in triplicate, and are presented as mean ± standard deviation (SD). For cytotoxicity studies, comparisons were performed among different concentrations and incubation periods (24, 48, and 72 h). Differences in tumor volume among treatment groups were also analyzed using ANOVA. The value of *p* ≤ 0.05 was considered statistically significant.

## Discussion

Dox has absorption peaks from 200 to 300 nm as revealed in Fig. 1a. In addition to AuNPs, the band intensity was reduced because of the presence of the band at 680–720 nm. The appearance of this new band can be attributed to interparticle interactions among AuNPs with added Dox and plasmonic coupling between adjacent AuNPs^31^. Aggregation can be confirmed by a color change from red to mauve and a minor increase in the AuNPs dimensions^32,33^ Accumulation of AuNPs may be caused by the substitution of citrate with Dox, resulting in the formation of the Dox@AuNPs NC^34,35^. Furthermore, the active groups of AuNPs exhibit higher electrostatic attraction for Dox compared to citrate groups, which explains the appearance of extra bands at longer wavelengths^36^. Fluorescence investigations confirm drug binding to AuNPs because Au metal effectively quenches the emission of many fluorophores^27,37^. The FTIR investigation further supports this, showing that AuNPs were enclosed with citrate groups, and Dox binding resulted in a reduction and shift in characteristic bands^38^. The high loading percentage of Dox on the surface of AuNPs can be attributed to hydrogen bonding as well as electrostatic interactions with free secondary hydroxyl and carboxylate groups present on the AuNP surface^39^. The preservation of the fluorescence signal indicates that the Dox structure remains intact upon loading^40^. The enhanced cytotoxicity of Dox@AuNPs NC compared to free Dox can be attributed to improved cellular uptake via endocytosis, which is more efficient than the passive diffusion of free Dox^41,42^. Nanocomposites entering cells through endocytosis allow for a higher local concentration of the drug in the cytoplasm, thereby enhancing the cytotoxic effect^43^. This mechanism aligns with the observed reduction in IC_50_ in MCF7 cells treated with Dox@AuNPs NC (Fig. 3), where half the concentration of free Dox achieved comparable cell killing. Our results were in accordance with previous work^25^ where they illustrated that the conjugation of doxorubicin to gold nanoparticles (Dox@AuNPs NC) significantly improves therapeutic results in breast cancer models, particularly the MCF7 cell line, by employing active endocytosis to overcome the challenges of free drug delivery. In contrast to free DOX, which depends on passive diffusion and is frequently vulnerable to multidrug resistance (MDR) efflux, the Dox@AuNPs NC (generally around 12 nm in size) penetrate cells more effectively, achieving a cytotoxic impact at considerably lower concentrations.

In vivo, the Ehrlich carcinoma-bearing mice demonstrated longer survival periods and greater tumor inhibition with Dox@AuNPs NC treatment (Fig. 4) than with free Dox. This may be partially attributed to the enhanced permeability and retention (EPR) effect, which known to facilitate nanoparticle accumulation in tumor tissues due to leaky vasculature and poor lymphatic drainage^44,45^. Additionally, the AuNPs’ large surface area and biocompatibility facilitate sustained drug release, potentially contributing to reduced systemic exposure compared to equivalent doses of free Dox^46^.

The current study reveals significant differences in FTIR spectral absorption frequencies and intensities between normal and malignant tissue. These variations indicate alterations in nucleic acid and lipid composition. Changes in protein absorption bands suggest modifications in protein content and conformation. During cancer development, fundamental cellular components undergo evident alterations in structure, conformation, and abundance^47,48^.

Collagen plays a significant role in biological processes including cell morphogenesis, proliferation, migration, differentiation, apoptosis, and carcinogenesis. Increased collagen content is observed in abnormal tissues. In this study, the band at 1034 cm^− 1^ corresponding to collagen was detected. Another difference between normal and malignant spectra was the glycogen-associated band at 1151 cm^− 1^ which was almost absent in malignant tissue, suggesting a marked reduction in glycogen levels during malignancy. Differences in symmetric and antisymmetric phosphate vibrations suggest increased DNA content in malignant tissue due to continuous replication in cancer cells. Protein composition can serve as a diagnostic indicator of the physiological state of cells. The decrease in protein signals in malignant tissue suggests altered energy metabolism to meet increased energy demands under malignant conditions^49,50^.

Finally, the combination of nanocarrier-mediated delivery, enhanced intracellular accumulation, and FTIR-confirmed biochemical normalization demonstrates improved therapeutic performance under the present experimental conditions compared to free Dox (Tables 1, 2). The results suggest that this platform may provide a basis for further preclinical investigation, including integration with photothermal or immunotherapy strategies in future studies^51–53^.

**Table 1:** The peaks positions and modes assignment of the main FTIR bands for normal, malignant tissues and malignant tissues after treatment with DOX coated AuNPs.

Wave number (cm^− 1^ )	Assignment
1034 cm^− 1^	Collagen
1065 cm^− 1^	C-O stretching of the phosphodiester and the ribose
1077 cm^− 1^	Symmetric phosphate [PO2 (sym)] stretching
1137 cm^− 1^	Oligosaccharide C-OH stretching band 2-Methylmannoside
1150 cm^− 1^	C-O stretching vibration
1151 cm^− 1^	Glycogen absorption due to C-O and C-C stretching and C-O-H deformation motions
1164 cm^− 1^	Mainly from the C-O stretching mode of C-OH groups of serine, threosine, & tyrosine of proteins)
1255 cm^− 1^	Amide III
1261 cm^− 1^	PO2– asymmetric (phosphate I)
1282 cm^− 1^	Amide III band components of proteins
1337 cm^− 1^	Collagen
1352 cm^− 1^	Stretching C-O, deformation C-H, deformation N-H
1359 cm^− 1^	Stretching C-O, deformation C-H, deformation N-H
1401 cm^− 1^	Symmetric CH3 bending modes of the methyl groups of proteins
1444 cm^− 1^	(CH2), lipids, fatty acids (CH) (polysaccharides, pectin)
1576 cm^− 1^	C = N adenine
1584 cm^− 1^	Ring C-C stretch of phenyl
1646 cm^− 1^	Amide I C = O, stretching C = C uracyl, NH2 guanine
1670 cm^− 1^	Amide I (anti-parallel ß -sheet) (C = C) trans, lipids, fatty acids
1755 cm^− 1^	(C = C) lipids, fatty acids
2678 cm^− 1^	Stretching N-H (NH3)
2928 cm^− 1^	Stretching C-H
2930 cm^− 1^	C-H stretching bands
3298 cm^− 1^	Amid A (N-H stretching)
3299 cm^− 1^	Amide A bands steming from N-H stretching modes in proteins and nucleic acids
3611 cm^− 1^	O-H & N-H stretching vibrations

**Table 2:** Comparison of IC_50_ values for Free DOX and DOX coated AuNPs at 24 h, 48 h, and 72 h.

Cell line	IC_50_					
	Free DOX			DOX –AuNPs nanocomposite		
MCF7	24 h	48 h	72 h	24 h	48 h	72 h
	3.7e^-5^M	1.04e^-6^M	6.7e^-7^M	1.5e^-5^M	8.6e^-7^ M	4.7e^-7^ M

### Study limitations

This study has several limitations. First, the in vivo evaluation was conducted in single tumor model using a limited size. Second, cardiotoxicity markers and long-term systemic toxicity were not directly assessed. Therefore, further studies involving multiple tumor models, extended safety profiling, and comprehensive mechanistic evaluation are necessary before clinical translation can be considered.

## Conclusion

Dox@AuNPs nanocomposites demonstrate enhanced anticancer efficacy both in vitro and in vivo, significantly reducing IC₅₀ in MCF-7 cells and prolonging survival in Ehrlich carcinoma-bearing mice. FTIR analysis revealed a shift of biochemical profiles toward normal tissue, suggesting improved tumor-associated biochemical modulation compared with free Dox. These findings indicate the translational potential of AuNP-based nanocarriers for safer and more effective chemotherapeutic delivery and provide a foundation for further development comprehensive safety and pharmacological evaluation.

## Data Availability

The dataset(s) supporting the conclusions of this article is(are) included within the article (and its additional file(s)).
